# Neighborhood greenspace visits and mental health: insights from mobility data across nine U.S. metropolitan areas

**DOI:** 10.3389/fpubh.2026.1731243

**Published:** 2026-02-06

**Authors:** Sarayu Chandra Mouli, Tong Zhang, Zhuo Chen, Sanjay Rajagopalan, Jay E. Maddock, Khurram Nasir, Weichuan Dong, Sadeer Al-Kindi

**Affiliations:** 1 Center for Health and Nature, Department of Medicine, Houston Methodist Research Institute, Houston Methodist Hospital, Houston, TX, United States; 2 School of Medicine, Case Western Reserve University, Cleveland, OH, United States; 3 Department of Cardiology, Houston Methodist, Houston, TX, United States

**Keywords:** greenspace visits, mental health, mobility, nature, spatial heterogeneity, urban environment

## Abstract

**Background:**

Access to greenspace supports mental health, but most studies focus on the presence of greenness rather than actual use. Fewer explore how the association may vary within city boundaries and suburban areas.

**Objective:**

We examined associations between neighborhood-level greenspace visits and poor mental health across nine U.S. metropolitan statistical areas (MSA), adjusting for Normalized Difference Vegetation Index (NDVI), demographics, and social vulnerability, and evaluated spatial heterogeneity.

**Methods:**

We integrated greenspace visits, CDC PLACES mental health estimates (2023), Social Vulnerability Index (2018), demographics (ACS 2010–2014), and NDVI (2021). Ordinary least squares (OLS) models were conducted for each metro and overall, adjusting for NDVI, SVI themes, and demographics. Multi-scale geographically weighted regression (MGWR) assessed spatial variation.

**Results:**

The sample included 13,152 tracts across nine MSAs (72.5 million residents). Poor mental health prevalence ranged from 12.9% (DC) to 18.7% (Houston), while visits ranged from 11.5 (Houston) to 33.2 (DC). In pooled models, more visits indicated better mental health (β = −0.029; *R*^2^ = 0.62). Metro-specific models showed strongest effects in Houston (β = −0.113; *R*^2^ = 0.716) and Atlanta (β = −0.096; *R*^2^ = 0.634), with weaker results in Philadelphia and DC. MGWR revealed strong urban-core associations in Houston, Atlanta, and Dallas, while peripheral tracts often showed null effects.

**Conclusions:**

Greenspace engagement, not just presence was linked to lower poor mental health across the metros, though associations varied by neighborhood. Promoting use may improve mental health, but interventions should account for local context and social vulnerability.

## 1 Introduction

Exposure to natural environments has long been recognized as an important determinant of mental health and wellbeing. In 1984, Ulrich showed that natural views accelerate physiological and emotional recovery from stress (Stress reduction theory/SRT) (1–3), while Kaplan and Kaplan proposed that nature replenishes depleted cognitive resources through Attention Restoration (ART) (4–6). These pioneering studies highlighted the psychological benefits of contact with nature in controlled settings. Building on this foundation, later epidemiological research began to document community wide and population-level benefits of greenspace, showing associations with reduced psychological distress, lower risk of depression, and improved wellbeing (7, 8). More recently, reviews and meta-analyses have consolidated this evidence, pointing to multiple pathways through which greenspace supports mental health, including stress reduction, opportunities for physical activity, social cohesion, and buffering of urban stressors. These pathways represent modifiable environmental determinants of mental wellbeing, central to public health programs, disease prevention and health promotion. Recent research has emphasized that these benefits may vary across populations and settings due to spatial and social heterogeneity in environmental exposure, reflecting differences in accessibility, quality, and local context (9, 10).

Much of the epidemiologic literature linking greenspace with mental health has thus far relied on static indicators of greenness, such as the Normalized Difference Vegetation Index (NDVI), to quantify exposure. While NDVI provides valuable information about the presence of vegetation, it does not capture whether residents engage with greenspaces (11). Because exposure opportunity does not necessarily equate to exposure behavior, actual use or engagement may better reflect realized environmental contact and potential health effects. Understanding this behavioral dimension of exposure is increasingly recognized as essential for translating environmental health evidence into equitable, actionable public health strategies. This gap has prompted calls for measures that capture actual utilization with greenspace, such as visits and usage patterns, which may be stronger predictors of realized health benefits. At the same time, studies suggest that variations in accessibility, safety, connectivity, and environmental quality influence how different populations experience greenspace (12). For instance, lower park density, limited transport connectivity, or perceived safety concerns can restrict use, while high-quality and well-connected greenspaces can promote engagement even in dense urban areas. Socio-environmental context therefore modifies the strength and direction of greenspace-health relationships observed across cities (10).

To address these gaps, this paper uses mobility-derived datasets to examine whether greenspace engagement, is associated with neighborhood-level prevalence of poor mental health across U.S. metropolitan areas. Leveraging analysis at a small geographic scale, we used estimates of mental health burden (CDC PLACES), social vulnerability index, population demographic characteristics, and NDVI to test the extent to which greenspace visits influence mental health, considering both engagement and context.

## 2 Methods

### 2.1 Study design

We conducted a cross-sectional ecological study at the census tract level across nine of the 10 largest U.S. metropolitan statistical areas (MSAs; hereafter referred to as metropolitan areas or “metros”). MSAs were defined using the U.S. Census Bureau's 2020 delineation of metropolitan and micropolitan areas. Miami was excluded due to the absence of tract-level health estimates in CDC PLACES. For each included metro, county Federal Information Processing Standards (FIPS) codes were obtained from the Census Bureau, and all constituent census tracts were selected. The number of counties per metro ranged from two (Phoenix, Los Angeles) to 22 (New York City), yielding a total of 13,152 census tracts across the nine study metros.

We compiled a comprehensive dataset by integrating information from multiple national sources that provide small area estimates or spatially continuous data (Table 1). These included health indicators, environmental exposures, social vulnerability indices, and demographic composition for each MSA at the census tract level. All data are publicly available, de-identified and contained no personally identifiable information and as such, institutional review board (IRB) approval and informed consent were not required.

**Table 1:** Data sources overview.

**Dataset**	**Source**	**Year of release**	**Variables used**
Mobility data	Advan Research	2021	Greenspace visits, Devices
CDC PLACES	CDC, RWJF	2023	MHLTH Crude Prevalence
Social Vulnerability Index	CDC/ATSDR	2020	RPL_THEME1-4 (SVI Themes)
Demographics	ACS	2014	% Female, % Black, Median Age

CDC, Centers for Disease Control and Prevention; RWJF, Robert Wood Johnson Foundation; ATSDR, Agency for Toxic Substances and Disease Registry; ACS, American Community Survey (conducted by the U.S. Census Bureau).

### 2.2 Data sources and variables of interest

Publicly available census tract-level health data were obtained from the CDC PLACES Project, which provides modeled estimates of chronic disease risk factors, health behaviors, and outcomes for U.S. census tracts. PLACES estimates are derived using small-area estimation methods that combine survey data from the Behavioral Risk Factor Surveillance System (BRFSS) with population demographics from the Census Bureau. The outcome was the prevalence of adults reporting ≥14 days of poor mental health in the past month, denoted as frequent mental distress in adults, a validated indicator for poor mental health used in population-level health surveillance.

Data on greenspace visits were obtained from Advan Research mobility traces (20), monthly and aggregated to the whole years of 2021. This dataset aggregates anonymized smartphone location data from millions of devices in the U.S. and provides visit counts to points-of-interest (POIs). Greenspace POIs were defined using the U.S. Park Serve database and OpenStreetMap land-use categories, restricted to public-access parks and recreational spaces. Greenspace visitation data for a given census tract were defined as the total number of visits to greenspace locations made by mobile devices whose owners were residents of that tract, divided by the total number of resident devices in the tract, defined as:


$$\begin{array}{l} \text{Greenspace visits }=\;\frac{Total\;Greenspace\;Visits\;by\;resident\;devices}{Number\;of\;Resident\;Devices} \end{array}$$

Device residency was determined using Advance Patterns' algorithm, which assigns a device's home census tract based on its long-term nighttime location. This normalization adjusts for variation in the density of devices observed by the mobility panel across geographic areas.

### 2.3 Covariates

Environmental and social covariates were selected to reflect contextual factors that may influence neighborhood mental health. Normalized Difference Vegetation Index (NDVI) was derived from 2021 VIIRS satellite imagery obtained through NASA's EarthData platform. 12 monthly raster files (8th day of each month in 2021) were downloaded, stacked, and averaged to create an annual mean index. Tract-level data was calculated using spatial overlays: census tract polygons (2010 TIGER/Line shapefiles) were used to extract mean NDVI values from the raster via zonal statistics. Values less than zero (representing water or blue spaces) were set to zero, resulting in our dataset that reflects census level vegetation presence across all our metropolitan areas. Finally, since the obtained data from 2021 primarily relied on 2020 census boundaries, we cross walked them to reassign 2020 bounded data to 2010 geographic areas.

Social vulnerability obtained from the CDC/ATSDR Social Vulnerability Index ((19) release), incorporates 15 census variables are grouped into four themes: socioeconomic status, household composition, racial/ethnic minority status and language, and housing/transportation. For this analysis, we included each of the four theme-specific indices. SVI values range from 0 to 1, with higher values indicating poorer social vulnerability.

The study also adjusted for demographics data, obtained from the American Community Survey (ACS, 2010–2014). Using tract-level data, we extracted three key variables: percentage of female residents, percentage of Black residents, and median age by census tract. These variables were queried and processed and merged into a complete dataset. All datasets were harmonized to the tract geography using 2010 U.S. Census boundaries and merged using GEOID identifiers. The final data frame, after merging all our individual datasets retained coverage for more than 95% of all tracts across the nine MSAs (Table 2). While data from Advan Research was obtained under a commercial license, all other datasets are publicly available. As all data were aggregated at the census-tract level and contained no personally identifiable information, institutional review board (IRB) approval and informed consent were not required.

**Table 2:** Census tract counts and data coverage across nine U.S. MSAs.

**MSA/Counties**	**CDC Places (*n*)**	**Mobility (*n*)**	**Demographics (*n*)**	**SVI (*n*)**	**NDVI (*n*)**	**Coverage (%)**
Atlanta (29)	948	948	882	948	948	93
Chicago (13)	2,167	2,167	2,119	2,167	2,147	97.8
DC (23)	1,264	1,264	1,209	1,264	1,254	95.6
Dallas (11)	1,309	1,309	1,223	1,309	1,309	93.4
Houston (10)	1,071	1,071	1,004	1,071	1,056	93.7
Los Angeles (2)	2,904	2,904	2,893	2,903	2,892	99.6
NYC (22)	4,138	4,138	4,097	4,138	4,138	99
Philadelphia (11)	1,464	1,464	1,434	1,464	1,464	98
Phoenix (2)	984	984	936	984	984	95.1

Values represent the number of census tracts within each metropolitan statistical area with available data from each source. “Tracts with Complete Data (%)” indicates the proportion of census tracts with non-missing data across all datasets used in the analysis (CDC PLACES, greenspace visits from Advan Research, NDVI, SVI, and ACS demographics).

### 2.4 Statistical analysis

All analyses were conducted at the census tract level within each of the nine metropolitan statistical areas (MSAs), relying on the official county-to-MSA crosswalk published by the Office of Management and Budget (OMB) and disseminated through the U.S. Bureau of Labor Statistics (BLS) (13). All analysis were conducted on R Studio 2025.05.0 Build 496. Prior to modeling, descriptive statistics were generated for all study variables to examine distributions and assess tract-level coverage across datasets.

For each MSA, an OLS regression model estimated the association between poor mental health prevalence and greenspace visits, adjusting for NDVI, SVI theme scores and demographic variables. Scatterplots were generated to visualize the associations.

Formally, the global OLS model can be expressed as:


$$\begin{array}{l} Yi\;=\;\beta 0\;+\;\beta 1\;GSi\;+\;\beta 2\;NDVIi\;+\;\beta 3\;SVIi\;+\;\beta 4\;Xi\;+\;\varepsilon i \end{array}$$

Where *Yi* denotes the prevalence of poor mental health in census tract I, Gsi represents greenspace visits, NDVIi captures the tract-level vegetation presence, SVIi represents SVI themes from the CDC/ATSDR, *Xi* denotes the demographic covariates, and ε*i* is the error term. Two supplementary OLS models were additionally conducted to evaluate the relative contributions of NDVI and greenspace visits by alternatively including and excluding each predictor alone and in combination while holding all other covariates constant. In addition, an overall OLS model pooling data from all nine MSAs was conducted. To explore spatial heterogeneity in these associations, we applied Multiscale geographically weighted regression (MGWR) for each MSA using the same model specification. MGWR relaxes the assumptions of traditional geographically weighted regression models (MGWR) by allowing regression coefficients to vary locally and at predictor-specific spatial scales. Adaptive bandwidths were selected for greenspace visitations automatically via cross-validation to balance local bias and variance, thereby defining the neighborhood contributing to each local estimate in a data-driven manner. Local coefficients of association (β) were estimated and visualized to examine spatial variation in the relationship between greenspace visits and poor mental health prevalence, using the formulation:


$$\begin{array}{l} Yi=\beta 0\left(ui,vi\right)+k\sum \beta k\;\left(ui,vi\right)Xik+\varepsilon i \end{array}$$

Together, the OLS and MGWR analyses provided a comprehensive view of both global (city-wide) and local (neighborhood-specific) relationships between greenspace engagement and mental health, supporting inference at scales relevant to urban public health and environmental planning.

## 3 Results

### 3.1 Descriptive patterns

**Figure 1:**
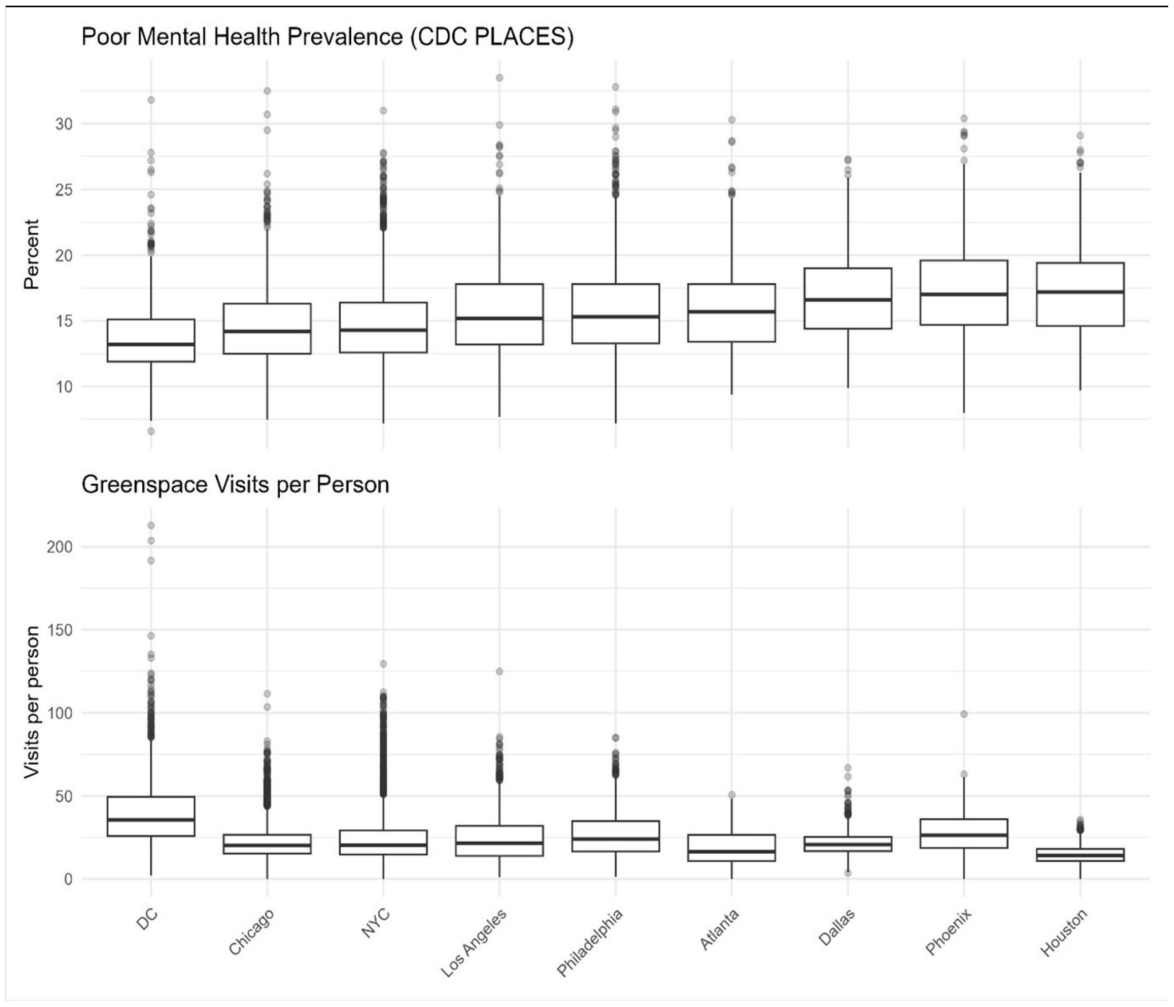
Distribution of poor mental health prevalence and greenspace visits across nine U.S. metropolitan statistical areas.

The 10 largest U.S. metropolitan statistical areas by population were initially selected for analysis. However, Miami was excluded due to the absence of census tract-level mental health estimates in CDC PLACES. The analytic sample included 13,152 census tracts across the nine MSAs, without the inclusion of Miami (Table 2). Tract counts ranged from 948 (Atlanta) to 3,951 (New York City). Figure 1 displays the distribution of poor mental health prevalence and greenspace visits across metros. Median prevalence of poor mental health ranged from 12.9% in Washington, DC to 18.7% in Houston, while median greenspace visits ranged from 11.5 in Houston to 33.2 in DC. Notably, these distributions appeared inversely patterned. Cities such as Houston showed both the highest prevalence of poor mental health and the lowest greenspace visits, whereas Washington, DC displayed the opposite trend. Other metros generally fell between these extremes, suggesting a consistent ecological association between greenspace engagement and mental health that is further explored in regression models.

### 3.2 OLS model results

**Figure 2:**
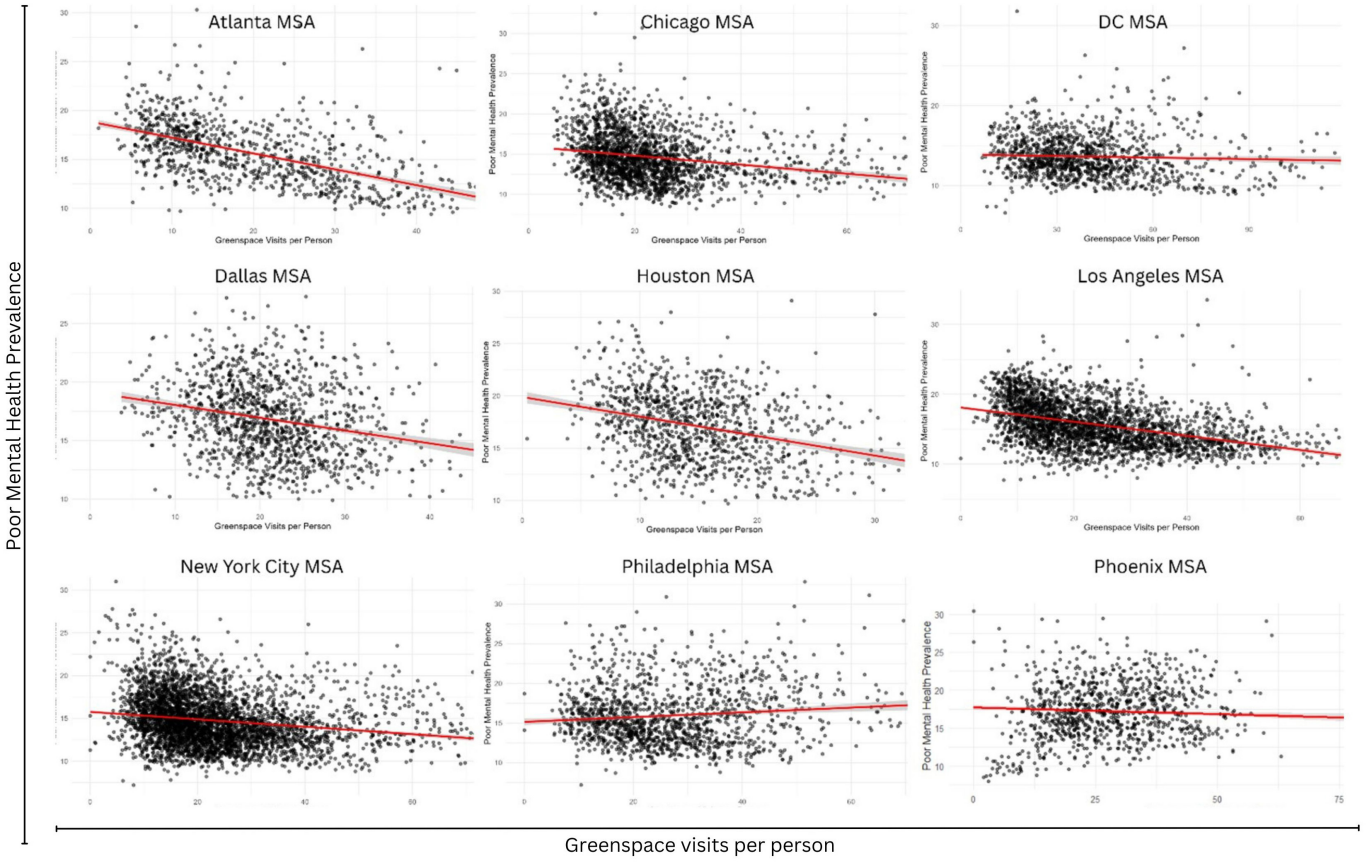
Association between greenspace visits and poor mental health prevalence by metropolitan area.

Adjusted ordinary least squares (OLS) models revealed significant negative associations between greenspace visits and poor mental health prevalence in most metros (Table 3, Figure 2). Higher levels of greenspace visitation were consistently associated with lower prevalence of poor mental health after adjusting for NDVI, and socioeconomic context, with effect sizes and model fit varying across MSAs. Model performance was strongest in several large metros, including Dallas, Washington DC, and Houston, where adjusted *R*^2^ values exceeded 0.70, indicating that our model captured a substantial proportion of tract-level variation in mental health outcomes.

**Table 3:** Adjusted OLS associations between greenspace visits and poor mental health prevalence by metropolitan area.

**MSA**	**Term**	**β (Greenspace visits)**	**CI levels [LL; UL]**	***p*-Value**	***R* ^2^**
Atlanta	Greenspace visits	−0.096	−0.11; −0.082	< 0.001	0.634
Chicago		−0.026	0.03; −0.0194	< 0.001	0.665
Washington DC		−0.007	−0.01; −0.004	< 0.001	0.796
Dallas		−0.024	−0.03; −0.014	< 0.001	0.857
Houston		−0.113	−0.13; −0.092	< 0.001	0.716
Los Angeles		−0.014	−0.02; −0.006	< 0.001	0.679
New York City		−0.018	−0.02; −0.012	< 0.001	0.550
Philadelphia		0.003	0.00; 0.009	0.289	0.851
Phoenix		−0.017	−0.03; −0.0071	< 0.001	0.765

Values shown represent the regression coefficient (β) for greenspace visits from multivariable ordinary least squares models. All models are adjusted for normalized difference vegetation index (NDVI), four Social Vulnerability Index (SVI) theme scores, and demographic covariates (percentage female, percentage Black, and median age). Confidence intervals and p-values correspond to the greenspace visits coefficient only.

To distinguish the effects of actual greenspace engagement (i.e., visitation) vs. greenspace availability (i.e., NDVI-derived coverage), we conducted sensitivity analyses in which models included each exposure separately and jointly (Supplementary Table S1). When modeled alone, greenspace visitation was consistently and inversely associated with poor mental health across most MSAs. In contrast, NDVI-only models showed substantial heterogeneity across metropolitan areas, with greater variability in both the magnitude and direction of associations and generally less stable model fit. When both measures were included simultaneously, model performance improved modestly in some MSAs but remained unchanged or decreased in others. Overall, these findings suggest that greenspace visitation captures much of the variation relevant to the mental health benefits of greenspace, beyond what is explained by the greenspace availability measure alone.

In pooled analyses across all nine metros, Model A (without Metro fixed effects) indicated that each additional greenspace visit was associated with a 0.037% lower prevalence of poor mental health (β = −0.037, *p* < 0.001l Supplementary Figure S1), with the model explaining just over half of the variance (*R*^2^ = 0.53; Supplementary Figure S2). When Metro fixed effects were added in Model B, model fit improved substantially (*R*^2^ = 0.62; Supplementary Table S2), reflecting city-level context accounted for additional variability. Relative to Atlanta (reference), Phoenix (+2.7 pp), Houston (+1.3), and Philadelphia (+1.2) exhibited higher adjusted baselines of poor mental health prevalence, while Washington, DC (−1.1) and Chicago (−0.7) were lower. In this specification, the association between greenspace visits and poor mental health remained robust though slightly attenuated (β = −0.029, *p* < 0.001). Taken together, these pooled models confirm higher greenspace engagement is consistently linked with better mental health outcomes across metropolitan areas, while also highlighting meaningful differences in baseline prevalence of both poor mental health and greenspace visits between cities (Supplementary Figures S3, S4). In our within-metro models, the strongest association were observed in Houston (β = −0.113, *p* < 0.001) and Atlanta (β = −0.096, *p* < 0.001), where each additional greenspace visit was associated with over a 0.1% lower prevalence of poor mental health. In contrast, Philadelphia (β = 0.003, *p* = 0.29) and Washington DC (β = −0.07; *p* < 0.001) showed weak or inconsistent associations. Model explanatory power varied, with *R*^2^ values ranging from 0.55 in NYC to 0.86 in Dallas, indicating robust model fit overall.

### 3.3 Spatial patterns (multiscale geographic weighted regressions)

**Figure 3:**
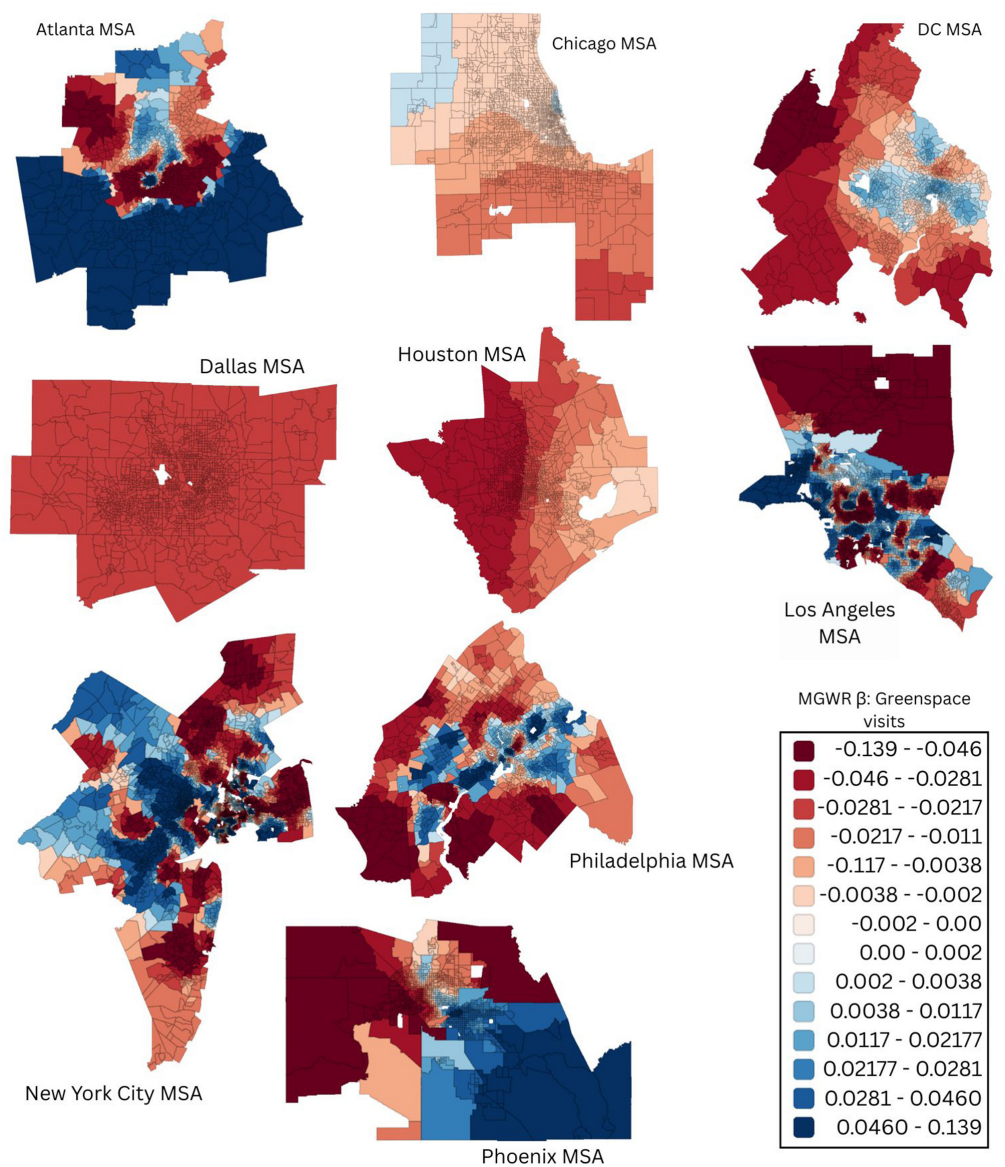
Multiscale geographically weighted regression (MGWR) coefficient surface showing the census tract-level association between greenspace visits and poor mental health across nine U.S. metropolitan areas. Blue indicates protective associations, white indicates near-null effects, and red indicates positive associations.

**Figure 4:**
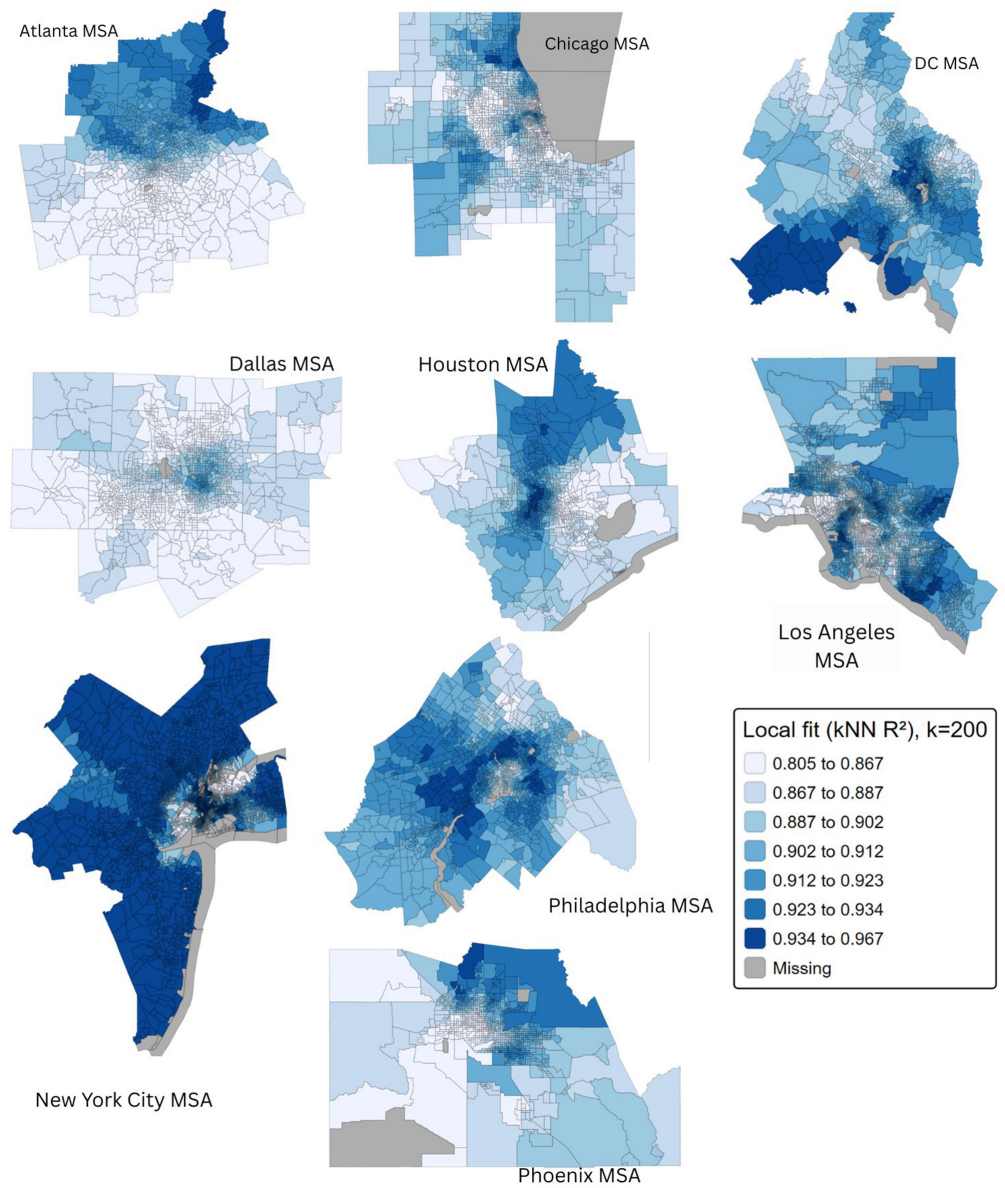
Local model fit (*R*^2^) surfaces for MGWR models across nine U.S. metropolitan areas, computed using a k-nearest-neighbor (*k* = 200) approach. Darker blue indicates higher local explanatory power of the MGWR model, while lighter shades indicate comparatively lower fit.

While OLS models identified metro-wide associations between greenspace visits and poor mental health prevalence, these estimates assume spatial homogeneity. To capture potential neighborhood-level variation, we applied multi-scale GWRs. The results of our MGWRs allowed to visualize the multiple spatial scales within each MSA. Our results revealed that the strength and direction of the greenspace-mental health association varied substantially across neighborhoods, with distinct patterns emerging in each MSAs (Figures 3, 4).

Because our aim was to test whether greater greenspace engagement corresponds with lower prevalence of poor mental health, inverse relationships (negative coefficients) are interpreted as favorable i.e., more greenspace visits is associated with improved mental health among the population. Conversely, positive coefficients suggest the opposite pattern, which we interpret as unfavorable. Across metropolitan areas, spatial heterogeneity followed a consistent contextual pattern rather than a purely geographic one. Stronger inverse associations between greenspace visits and poor mental health were concentrated in dense, higher socially vulnerability urban neighborhoods, whereas lower-density, lower-vulnerability suburban and exurban tracts generally exhibited weaker to mild or inconsistent relationships.

In Atlanta, the strongest beneficial relationships were seen in the southern tracts like Fulton and DeKalb Counties, areas that are characterized by historically higher social vulnerability, while northern suburban tracts with lower vulnerabilities showed weaker or no associations (14). Houston exhibited a similar vulnerability-stratified pattern with strong inverse associations concentrated in the urban core and weaker associations in outer suburban tracts (Montgomery and Fort Bend Counties). In Dallas, favorable associations were concentrated in socially vulnerable urban tracts, while outer suburban areas exhibited weaker effects. Los Angeles and Chicago showed more mixed patterns.

Patterns in Los Angeles and Chicago were more heterogeneous but followed similar contextual gradients. Tracts with greater access to large, centrally located public parks showed stronger associations, whereas lower-density coastal and valley areas showed minimal effects. In Chicago, tracts along the lakefront and in the central city In Chicago, tracts along the lakefront and in the central city.

New York City showed strong inverse associations in dense tracts surrounding major public greenspaces, particularly in parts of Manhattan, Brooklyn, and Queens, while outer borough tracts exhibited weaker or unfavorable associations. Philadelphia represented a notable exception in the overall association pattern of our analysis. Although some tracts near Fairmount Park showed favorable relationships, many surrounding neighborhoods exhibited weak or unfavorable associations, consistent with the null findings observed in metro-wide OLS models. In Washington, DC, associations were generally weak across the district, with modest localized effects near major parks like Rock Creek but minimal effect in the Eastern neighborhoods.

Local models were estimated for each census tract using an adaptive spatial kernel, such that the number of neighboring tracts contributing to each local regression varied according to tract density. The optimal bandwidth was selected automatically by minimizing the cross-validation (CV) score, balancing local bias and variance.

To complement the metro-specific models, we also conducted pooled analyses across all nine MSAs (Supplementary Table S2). In Model A, without accounting for variations between MSAs, greenspace visits were inversely associated with poor mental health (β = −0.037, *p* < 0.001; *R*^2^ = 0.53). After adding Metro fixed effects in Model B, the association persisted but was modestly attenuated (β = −0.029, *p* < 0.001), while model fit improved considerably (*R*^2^ = 0.62), underscoring the importance of accounting for city-level differences. These pooled results confirm the robustness of the overall relationship, even as the metro-specific models highlight substantial heterogeneity in both magnitude and direction of effects.

Across metros, a consistent urban-suburban gradient was observed. MGWR local fit surfaces were computed using a k-nearest-neighbor (kNN) approach, where local *R*^2^ values were estimated by comparing observed and fitted outcomes within a fixed neighborhood (*k* = 200) around each census tract. The *R*^2^ maps showed consistently high explanatory power across all metros (local *R*^2^ ≥ 0.80), with stronger fit in dense urban neighborhoods and slightly lower but still robust fit in peripheral areas (Figure 4). This pattern suggests that greenspace engagement may be particularly salient for mental health in high-density areas, where exposure to stressors and social vulnerability is greater but public park access is more frequent and well connected. In contrast, suburban and exurban contexts may have larger quantities of greenspace, but less public transit, more fragmented accessibility, and a reliance on private yards, which may limit the degree to which greenspace engagement translates into measurable mental health benefit. Taken together, these findings indicate that the mental health benefits associated with greenspace engagement are highly context-dependent, with the strongest associations occurring in dense, socially vulnerable urban neighborhoods patterns that are obscured in city-wide average estimates.

## 4 Discussion

This study examined the relationship between greenspace engagement and poor mental health prevalence across nine major U.S. metropolitan areas. Using mobility-derived visits as a measure of greenspace usage, we found that greater greenspace engagement was generally associated with lower prevalence of poor mental health at the census-tract level. Our findings are consistent with prior research emphasizing the benefits of greenspace on mental health (7, 8). More importantly, our research extends prior work by demonstrating that these benefits are contingent not merely on the presence of greenspace but also on actual engagement with it. Notably, the association remained significant in most cities after adjusting for NDVI, demographic characteristics, and social vulnerability, with particularly strong effects in Houston and Atlanta. Sensitivity analyses comparing alternative greenspace specifications (visitation-only and NDVI-only models) yielded directionally results that varied from our fully adjusted models, with some cases showing staggeringly extreme estimates, highlighting the importance of jointly accounting for both greenspace presence and use. The improved model fit following these adjustments suggests that the mental health benefits of greenspace engagement are shaped by underlying social and environmental vulnerability, aligning with prior literature (10, 12).

The MGWR analysis demonstrates that associations between greenspace engagement and poor mental health are not only spatially heterogeneous, but also operate at multiple spatial scales, bringing out the importance of neighborhood context in shaping healthy environments. By allowing the influence of greenspace visits to vary locally while accounting for spatial autocorrelation, our MGWR provides a more granular representation of how behavioral exposure to greenspace relates to mental health than global or single-scale local models. This multiscale framework clarifies how global citywide estimates (average associations) may conceal substantial neighborhood-level heterogeneity, particularly in socially diverse metropolitan regions.

Stronger inverse associations were consistently observed in dense, socially vulnerable urban neighborhoods, while suburban and peripheral areas exhibited weaker or null relationships. For instance, in Houston, poor mental health prevalence was highest in Harris County's urban core, overlapping with tracts where greenspace visits were relatively low, while wealthier outer suburbs such as Fort Bend and Montgomery Counties exhibited weaker associations. In Washington DC, the strongest relationships clustered around Rock Creek Park, whereas peripheral counties like Culpeper and Fauquier had generally lower prevalence of poorer mental health and fewer visits, which may help explain why associations there were weaker or null. Areas close to major urban greenspaces (UGS), such as downtown Los Angeles near Griffith Park, and neighborhoods around Central Park in New York City, exhibit stronger relationships. This pattern suggests that greenspace engagement may be most beneficial where psychosocial stressors are higher and public greenspaces are more accessible and routinely used. In contrast, suburban and exurban areas often contain more abundant but less publicly accessible or less frequently used greenspace (e.g., private yards or fragmented parks), which may limit the extent to which greenspace engagement translates into measurable mental health benefits. The unexpected patterns observed in Philadelphia further indicate that unmeasured contextual factors such as greenspace quality, safety, or cultural relevance may modify these associations, reinforcing the need for context-sensitive interpretations of greenspace- mental health relationships.

In contrast to dense urban neighborhoods where greenspace use appears most strongly associated with better mental health, peripheral areas often contain greenspaces that is either less accessible or less conducive to regular public use, despite being more abundant such as private yards or isolated parks. In these contexts, lower visitation may partly explain weaker associations. For example, Rieves et al. (18) found that perceived greenspace can differ from objective vegetation measures like NDVI, and both are linked to mental health in distinct ways (15). In some contexts, perceived safety or poor quality of the greenspace may reduce use altogether, resulting in weak or null associations. In others, greenspaces may still be used due to proximity, or routine travel patterns but may not confer mental health benefits if the experience is stressful, unsafe, or culturally misaligned. This distinction may explain why some tracts exhibited weak or unfavorable associations despite measurable visitation, but fails to translate into better mental health outcomes, as suggested in prior literature (16). In communities where greenspace is scarce, increasing both the supply and activation of greenspaces may directly improve mental health outcomes. These findings suggest that investments in greenspace infrastructure should be paired with interventions that promote and sustain consistent usage across communities, with a focus on residential access. Urban planning analyses may benefit from considering both vegetation and behavioral exposure metrics, a shift that aligns with emerging “smart city” approaches leveraging big data, mobility traces, to better understand how residents interact with urban environments (17). At the same time, simply increasing the presence of greenspace is insufficient, particularly if communities cannot/does not use them due to safety, irrelevance to their needs. Context-sensitive engagement strategies that integrates usage to needs may be necessary to ensure that mental health benefits of greenspace are realized broadly. Prior conceptual reviews also highlight the benefits of greenspace engagement for vulnerable populations, explaining the differences in accessibility or utilization (12). Integrating greenspace strategies into broader health equity frameworks, including neighborhood audits and participatory design approaches represents a critical step toward improving social and environmental determinants of health. By linking behavioral mobility data with neighborhood health and vulnerability indicators, this study bridges environmental epidemiology and public health practice, informing prevention-oriented urban design and community-level health promotion efforts that advance broader goals of health and resilience and adaptive wellbeing.

### 4.1 Strengths and limitations

This study offers several strengths that advance understanding of greenspace and mental health. By analyzing nine of the largest U.S. metropolitan areas, we provide one of the few multi-city perspectives on neighborhood-level associations, capturing both commonalities and differences across diverse urban-suburban contexts. The use of mobility traces to measure greenspace visits represents an important innovation, moving beyond static measures of greenness toward actual engagement, more closely tied to realized health benefits. Finally, the application of MGWR revealed spatial heterogeneity within each city, that global models cannot detect, offering insight into the highly localized nature of greenspace-health relationships. Together, these strengths provide a robust and scalable framework for future research at the intersection of urban design, behavioral data, and population health.

A few limitations must be noted. First, the cross-sectional design precludes causal inference, and results should be interpreted as associations rather than effects. Second, analyses were conducted at the census-tract level, raising the possibility of ecological fallacy when inferring individual outcomes; moreover, survey-based health estimates may themselves be biased if participation or reporting varies across neighborhoods, which may help explain unfavorable associations in some areas. Third, mobility data are subject to smoothing and potential underrepresentation of certain populations, which could bias estimates of greenspace use. Additionally, our dataset consists of cumulative annual exposures and outcomes, based on CDC estimates and Advan research, which may not be perfectly aligned. Future work should examine multi-year mobility trends. Finally, measures of greenspace quality, amenities, and safety were not included, factors that as previously mentioned could account for some of the spatial heterogeneity observed.

Future research should adopt longitudinal designs to assess temporal dynamics in greenspace use and mental health. For example, seasonality considering weather and post-COVID19 effects may impact the patterns of outdoor activities and social vulnerability factors. Incorporating finer measures of greenspace quality and accessibility, as well as equity-focused audits, would strengthen the evidence base for interventions. Linking mobility data with health services, such as nature-based prescription programs could further clarify how greenspace can be operationalized in public health practice.

In summary, this study provides new evidence that greenspace engagement is associated with mental health outcomes in U.S. metropolitan areas, and the strength of associations vary across space and are strongly conditioned by social vulnerability. These results highlight the importance of moving beyond measures of greenness to consider actual use, and of pairing greenspace planning that address the social and structural contexts shaping mental health.

## Data Availability

The data analyzed in this study is subject to the following licenses/restrictions: With the exception of the greenspace visits data obtained from Advan Research (formerly SafeGraph), all datasets used in this study are publicly available from their respective repositories: CDC PLACES (https://www.cdc.gov/places/), CDC/ATSDR Social Vulnerability Index (https://www.atsdr.cdc.gov/placeandhealth/svi/index.html), and NDVI from NASA EarthData VIIRS (https://earthdata.nasa.gov/). Requests to access these datasets should be directed to salkindi@houstonmethodist.org.
