# Supplementary material for: Neighborhood greenspace visits and mental health: insights from mobility data across nine U.S. metropolitan areas
Source: Front Public Health. 2026 Feb 6;14:1731243. doi: 10.3389/fpubh.2026.1731243 (PMC12922516; doi:10.3389/fpubh.2026.1731243)
Supplement: Supplementary file 1 [file Table_1.docx]

Supplementary Material

# Supplementary Figures and Tables

## Supplementary Figures

### Figure S1: *Model A: Pooled Analysis across nine MSA, without Metro Effects*


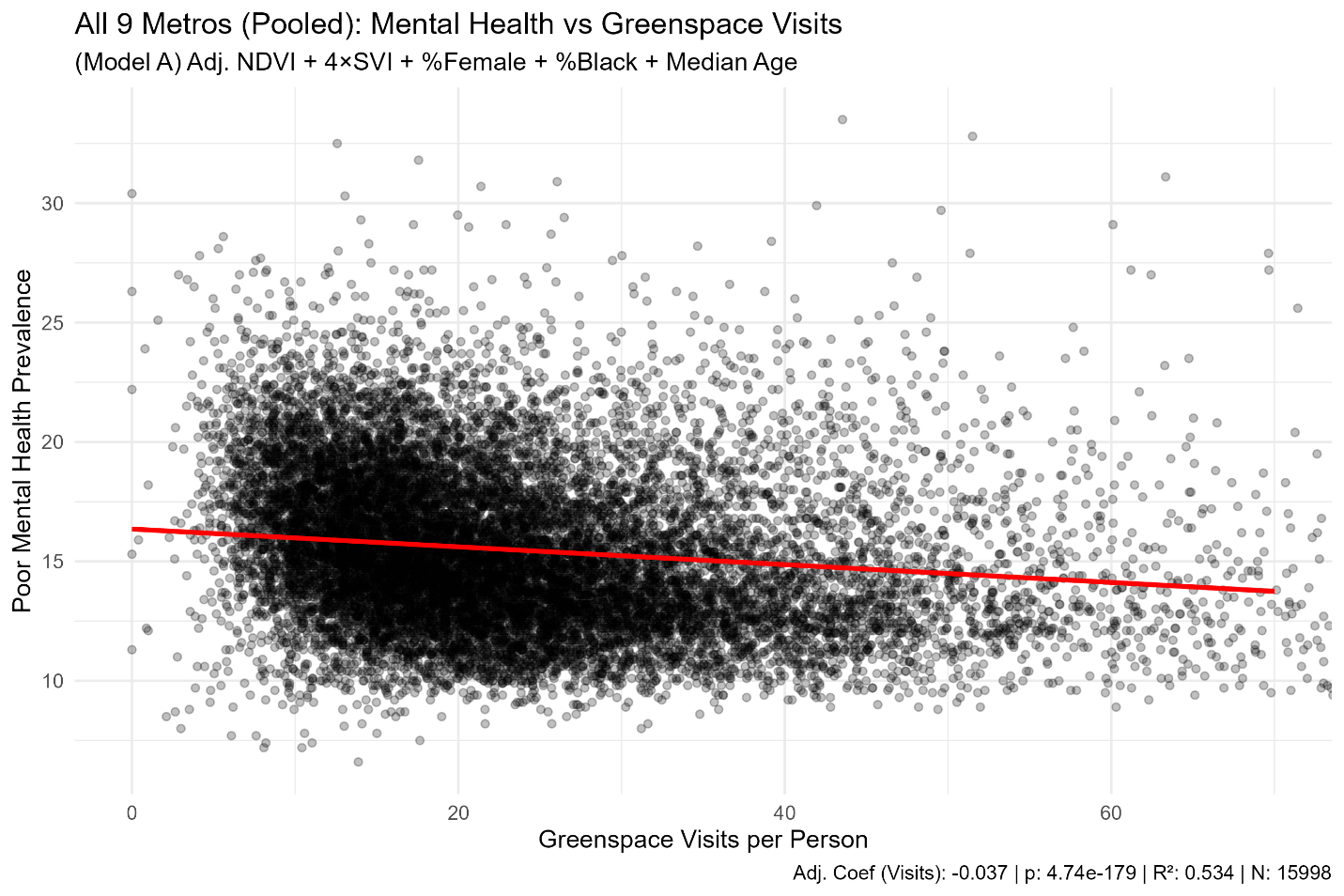


***Supplemental Figure S1: Model A: Pooled Analysis across nine MSA, without Metro Effects***

*
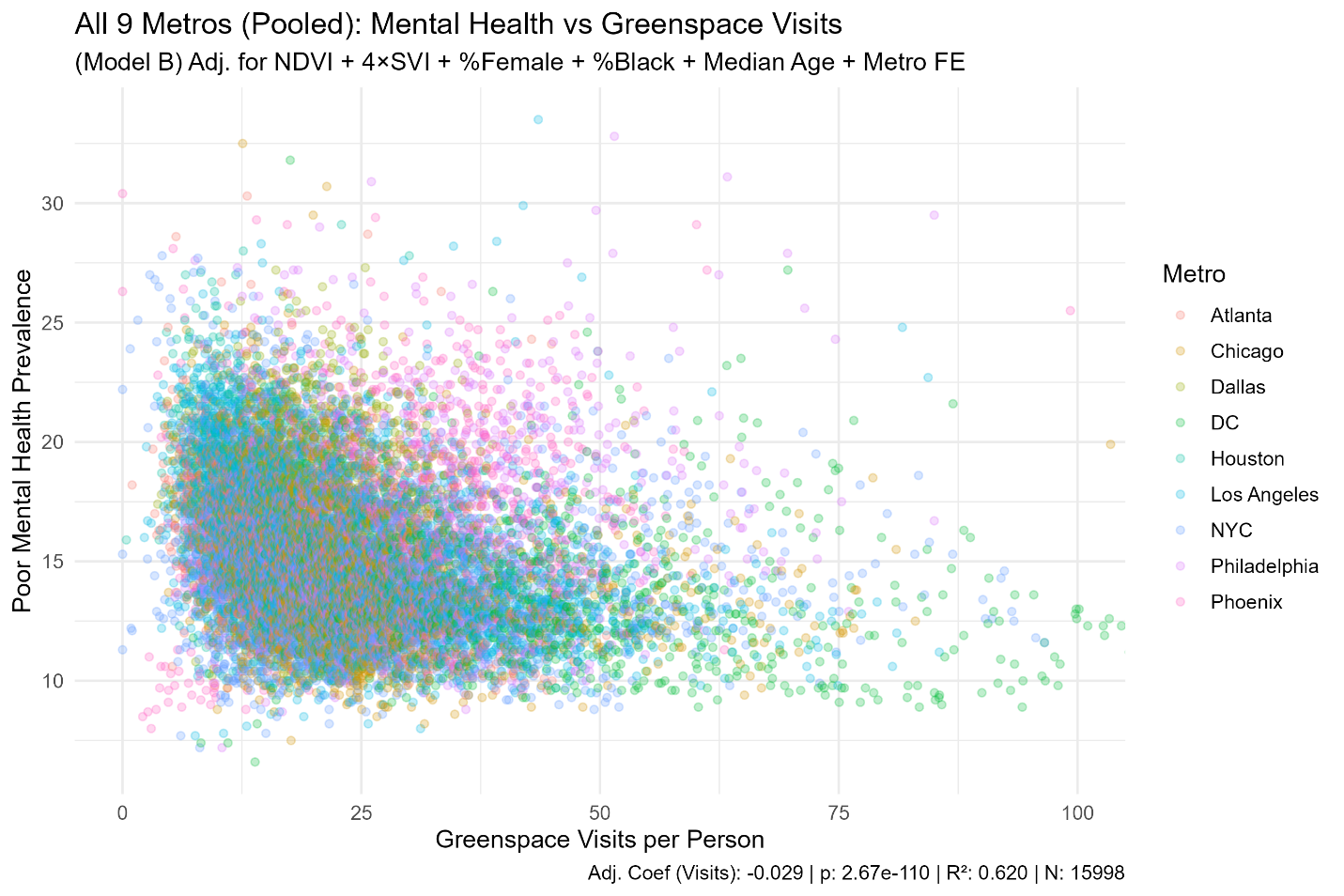
*

***Supplemental Figure S2: Model B: Pooled Analysis across nine MSA, with Metro Effects***

*
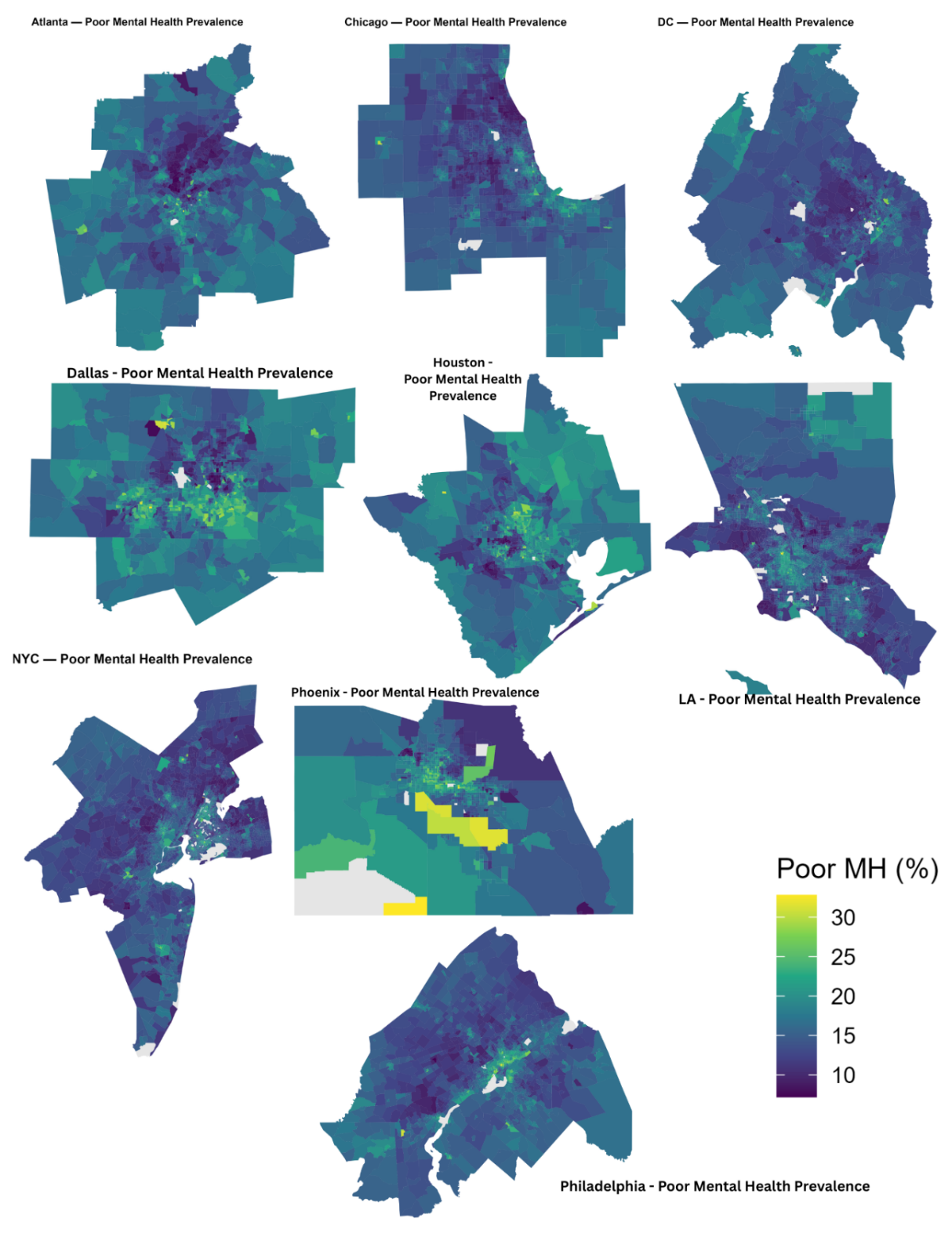
*

***Supplemental Figure S3: Spatial Variance of Poor Mental Health Prevalence by MSA***

*
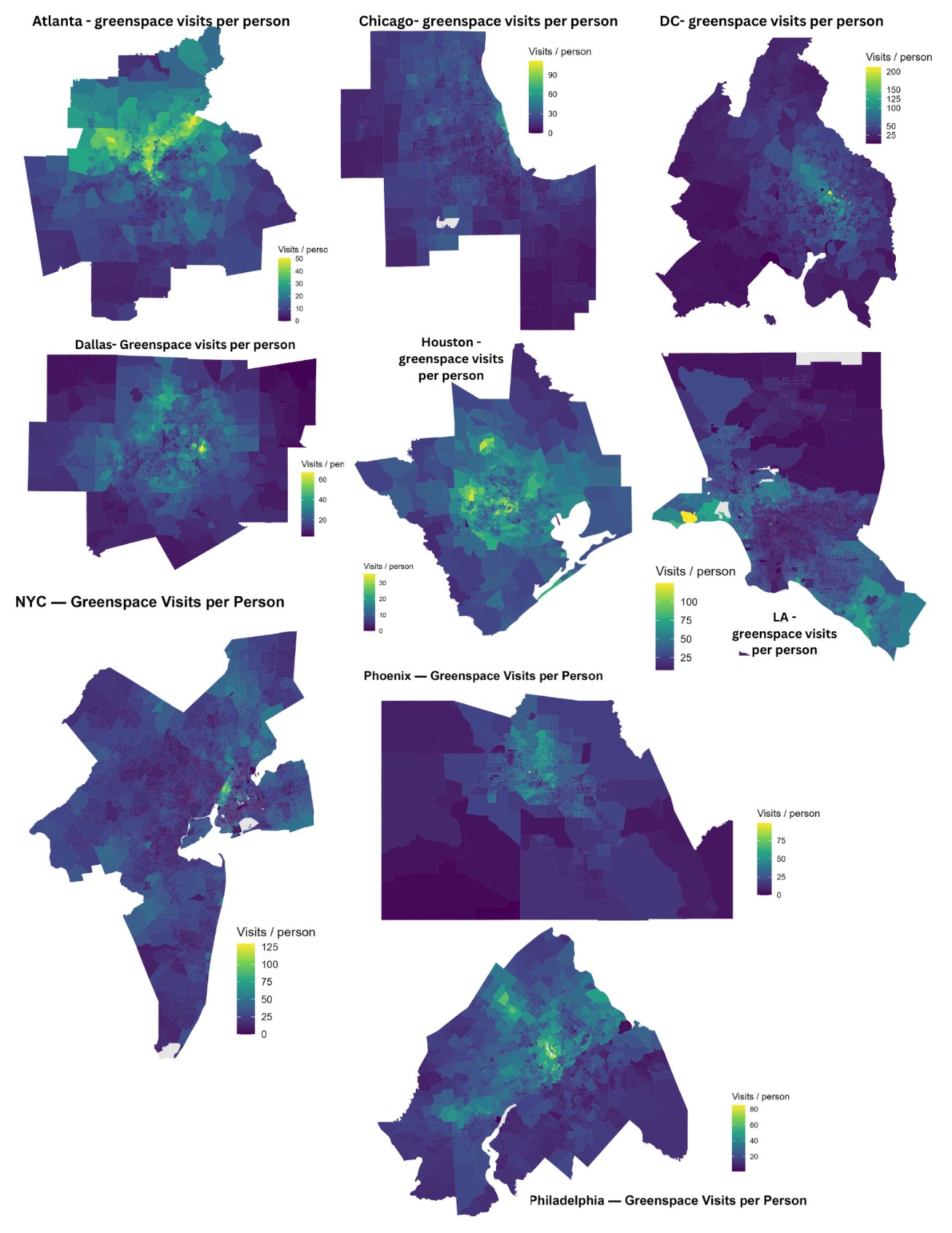
*

***Supplemental Figure S4: Spatial Variance of Greenspace visits per person by MSA***

***Supplemental Table S1: Alternative OLS model specifications examining visitation-based and vegetation-based greenspace exposures.***

| Metro | Model 1:  β (NDVI only) | R-Sq | Model 2:  β (Greenspace visits only) | R-Sq |
| --- | --- | --- | --- | --- |
| Atlanta | -0.114 | 0.556 | -0.094 | 0.630 |
| Chicago | 2.671 | 0.655 | -0.031 | 0.662 |
| DC | 0.813 | 0.7914 | -0.007 | 0.797 |
| Dallas | 0.0701 | 0.853 | -0.023 | 0.856 |
| Houston | -0.680 | 0.683 | -0.115 | 0.711 |
| Los Angeles | -3.4357 | 0.676 | -0.021 | 0.672 |
| NYC | -2.262 | 0.543 | -0.0146 | 0.549 |
| Philadelphia | -2.402 | 0.850 | 0.0067 | 0.847 |
| Phoenix | -3.4702 | 0.760 | -0.0161 | 0.756 |

****Model 1: Greenspace visits only (Adjusted for SVI, Demographics)***

****Model 2: NDVI only (Adjusted for SVI, Demographics)***

***Supplemental Table S2: Overall Pooled OLS summary, with and without MSA***

| term | β (Greenspace visits per person) | p-val (CI levels) | R-sq |
| --- | --- | --- | --- |
| Greenspace  Visits | -0.03728 | <0.01 (-0.039; -0.034) | 0.534173 |
|  | -0.02857 | <0.001 (-0.031; -0.0260) | 0.620478 |
| Chicago | -0.73512 | <0.001 (-0.904; -0.565) |  |
| Dallas | 1.268046 | <0.01 (1.091; 1.444) |  |
| DC | -1.08297 | <0.01 (-1.266; -0.899) |  |
| Houston | 1.330522 | <0.01 )1.148; 1.512) |  |
| Los Angeles | 0.710226 | <0.01 (0.544; 0.875) |  |
| NYC | -0.39041 | <0.01 (-0.556; -0.224) |  |
| Philadelphia | 1.21012 | <0.0001 (1.029; 1.390) |  |
| Phoenix | 2.688387 | <0.0001 (2.489; 2.88) |  |
